# Heterogeneity of monosomy 3 in fine needle aspiration biopsy of choroidal melanoma

**Published:** 2013-09-07

**Authors:** Melinda Y. Chang, Nagesh P. Rao, Barry L. Burgess, Lariza Johnson, Tara A. McCannel

**Affiliations:** 1 Department of Ophthalmology, University of California, Los Angeles, CA; 2 Department of Laboratory and Pathology Medicine, University of California, Los Angeles, CA; 3 Jules Stein Eye Institute, University of California, Los Angeles, CA

## Abstract

**Purpose:**

To report on the heterogeneity of monosomy 3 in a fine needle aspiration biopsy obtained transsclerally from choroidal melanoma for prognosis.

**Methods:**

All clinical records for patients who had been diagnosed with choroidal melanoma and underwent iodine-125 plaque brachytherapy with intraoperative transscleral fine needle aspiration biopsy from January 2005 to August 20, 2011, and who had a positive result for monosomy 3 according to fluorescence in situ hybridization as reported by clinical cytogenetics testing were collected. Patient age and sex, total number of cells evaluated and number of cells positive for monosomy 3, tumor size, and metastatic outcome were recorded for each patient.

**Results:**

A positive result for monosomy 3 was reported in 93 patients who underwent transscleral fine needle aspiration biopsy. Two patients were lost to follow-up immediately post-operatively, and the remaining 91 patients were included in this study. The mean number of cells evaluated in the biopsy was 273 (range 28 to 520). The mean percentage of cells positive for monosomy 3 was 62.9% (range 4.7%–100%). The mean tumor height was 5.91 mm (range 1.99 to 10.85 mm). Larger tumors were associated with a higher percentage of cells positive for monosomy 3. During the average follow-up interval of 28.9 months (range 3–76 months), choroidal melanoma metastasis developed in 18 (20%) patients. Patients whose tumors had 1%–33% of cells positive for monosomy 3 had a significantly lower risk of metastasis-related death compared to patients whose tumors harbored a higher percentage of monosomy 3 (p=0.04).

**Conclusions:**

Cytogenetic heterogeneity of fluorescent in situ hybridization for monosomy 3 exists in a biopsy sample. Larger tumors were more likely to have a higher percentage of monosomy 3 positive cells in the sample. Furthermore, patients whose tumors had more than 33% of cells positive for monosomy 3 had a poorer prognosis than patients whose tumors had lower percentages of monosomy 3.

## Introduction

Choroidal melanoma is the most common primary intraocular tumor in adults [1]. The 15-year risk of metastatic disease is approximately 50%, with the median survival after metastasis less than 6 months [2,3]. The risk factors for metastatic disease include the clinical, histologic, and cytogenetic characteristics of the primary tumor. The most robust predictor of metastatic disease to date is the loss of one copy of chromosome 3 [4].

Because of the strong correlation between chromosome 3 status and metastatic outcome, intraoperative fine needle aspiration biopsy (FNAB) may be performed during brachytherapy or immediately following enucleation. This method is reliable and safe for obtaining representative samples of tumor tissue to provide molecular prognosis [5-7].

Previous studies have established that, among uveal melanomas positive for monosomy 3, this cytogenetic alteration may not be present in every tumor cell [8-14]. The heterogeneity of monosomy 3 in uveal melanomas raises the question of what percentage of monosomy 3 in the tumor is necessary to constitute a poor outcome. A recent review reported that the cutoff point used for determining the presence of monosomy 3 varies widely in the literature, from 5% to as high as 60% [15]. There is currently no accepted threshold value for labeling a specimen monosomy 3 positive. However, Bronkhorst et al. [8] recently reported that a monosomy 3 cutoff value of 30% was the most robust predictor of metastasis-related death, although cutoff values of 5% and 50% also predicted poor prognosis. Most recently, van den Bosch et al. [16] reported on the heterogeneity of monosomy 3 in sections of enucleated specimens. Tumors were stratified into three groups based on the percentage of monosomy 3, and prognosis was incrementally worse with higher percentages of monosomy 3. Although one might extrapolate from this that the situation in a fine needle biopsy would yield a similar result regarding metastasis, no data to date have supported an increased risk of metastasis with a higher percentage of monosomy 3 in a fine needle biopsy.

Although larger tumor size correlates with the presence of monosomy 3 [17,18], the relationship between tumor size and percentage of monosomy 3 in the tumor has not been established. We hypothesized that larger tumors may have a higher percentage of cells with monosomy 3, because they are more advanced and the cells may therefore have had more opportunity to lose a copy of chromosome 3.

Most previous studies on the prognostic value of monosomy 3 in uveal melanoma used tumor samples obtained during enucleation [4,8,16]. Although this procedure has yielded important information on the poor prognosis of tumors positive for monosomy 3, this procedure is not directly applicable to patients who have monosomy 3 positive tumors discovered with FNAB during brachytherapy, as patients selected for brachytherapy generally have smaller and less advanced tumors. We present data on the prognostic value of monosomy 3 in patients undergoing globe-sparing therapy.

We reviewed the charts of patients with uveal melanomas who underwent brachytherapy with transscleral FNAB and were positive for monosomy 3 according to fluorescence in situ hybridization (FISH). Based on these data, we evaluated whether there was an association between 1) percentage of monosomy 3 and metastatic disease and 2) tumor height and percentage of monosomy 3.

## Methods

The study was performed in accordance with the U.S. Health Insurance Portability and Accountability Act (HIPAA) of 1996 and was approved by the Office of the Human Research Protection Program (Institutional Review Board) of the University of California, Los Angeles. The records of all patients who gave informed consent and had a clinical diagnosis of choroidal melanoma who underwent intraoperative transscleral FNAB during plaque brachytherapy at the Jules Stein Eye Institute, University of California, Los Angeles, between January 1, 2005, and August 20, 2011, were reviewed. All patients whose tumors were positive for monosomy 3 according to FISH were included in this study, excluding patients who were lost to follow-up immediately post-operatively.

Diagnosis of choroidal melanoma was established with comprehensive ophthalmic examination, ultrasonography, and ﬂuorescein angiography. Systemic evaluation by an oncologist or internal medicine specialist revealed no evidence of melanoma metastasis or other active primary cancer, and psychological support was offered by a clinical psychologist or social worker with expertise in choroidal melanoma [19].

Iodine-125 plaque surgery with FNAB was performed by a single surgeon (TAM). Details of FNAB and radioactive plaque placement have been described elsewhere [7,19-22]. Briefly, FNAB was performed with a 30-gauge needle via a tangential transscleral approach. Examination with binocular indirect ophthalmoscopy was performed immediately after FNAB, and the plaque was sutured in place. Optimal plaque position was achieved with intraoperative ultrasonography [23].

Biopsy specimens were processed for cytopathologic analysis and cytogenetic analysis as described in a previous report [22]. In general, the first biopsy specimen was immediately smeared on glass slides, fixed in ethanol, and stained with hematoxylin–eosin. A sample from an additional biopsy and/or residual material in the initial needle was rinsed in culture medium for cytogenetic analysis. Indeterminate samples were later tested with immunohistochemistry for the human melanoma black-45 antibody.

Chromosome 3 status was evaluated at the University of California, Los Angeles Clinical Cytogenetics Laboratory, a standardized CLIA-approved laboratory with a cytogeneticist experienced in interpreting and reporting FISH results for uveal melanoma. Cells collected for cytogenetic analysis were gently spun down in a sterile conical tube and resuspended in Rosewell Park Memorial Institute 1640 (RPMI-1640) media (Gibco [Invitrogen], Carlsbad, CA) supplemented with antibiotics and 10% bovine serum (Irvine Scientific, Santa Ana, CA). The cultures were observed for appropriate growth and mitotic activity, and cytogenetic studies were performed on short-term cultures prepared according to standard protocols. For FISH analysis, a directly labeled centromeric probe specific for chromosome 3, CEP-3 Spectrum Orange (Vysis, Downers Grove, IL), was used to assess monosomy or disomy. This probe was hybridized to fixed cultured cells following the manufacturer’s protocol (Abbott-Vysis, Des Plaines, IL). Hybridization signals were counted by hand in nonoverlapping nuclei of cells under a fluorescence microscope (Axiophot, Carl Zeiss Mikroskopie, Jena, Germany) equipped with a triple filter (diamino-2-phenylindole dihydrochloride/fluorescein isothiocyanate/Texas Red). When any cells were found to contain only one copy of chromosome 3, the sample was considered positive for monosomy 3.

Patients were evaluated post-operatively according to a standardized schedule. Follow-up visits were scheduled at 1 week, 1 month, 3 months, and 6 months after brachytherapy, and every 6 months thereafter. Starting at 3 months, A- and B-scan ultrasonography was performed at each visit to evaluate tumor response to brachytherapy. Patients were also instructed to undergo biannual systemic evaluation for metastatic disease, which included liver function testing and abdominal imaging with ultrasound, computed tomography, magnetic resonance imaging, or combined positron emission tomography/computed tomography at a minimum of once per year. The patient’s primary care physician or medical oncologist was contacted twice per year to reinforce the investigations for systemic surveillance.

Patient charts were retrospectively reviewed, and the following data were collected: demographic information, tumor parameters, date of brachytherapy surgery, FNAB cytopathology, choroidal melanoma metastatic disease and/or tumor-related death, monosomy 3 status according to FISH analysis, and last date of follow-up. The online Social Security Death Index was searched using patients’ names and birthdates to identify and confirm deceased patients. In the case of patients who were found to be deceased using this index, the patients’ families were contacted to determine whether the cause of death was metastatic disease. Length of follow-up was determined by calculating the number of days between the date of the brachytherapy surgery and the last date of follow-up. Patient data were tabulated using Excel (Microsoft Ofﬁce Excel 2003; Microsoft, Redmond, WA) and analyzed using Excel and MedCalc (MedCalc version 12.5.0.0, MedCalc Software bvba, Ostend, Belgium).

The Pearson correlation coefficient was used to evaluate the relationship between the percentage of monosomy 3 and tumor height. Kaplan–Meier survival curves were generated for analyzing metastasis-related survival by using the log-rank test. A p value of less than 0.05 was considered statistically significant.

## Results

Between January 2005 and September 2011, 93 patients with uveal melanoma who had undergone tumor treatment with iodine-125 plaque brachytherapy were identified whose FISH result from FNAB was positive for monosomy 3. Two patients were lost to follow-up immediately post-operatively and were excluded from this study. The patient and tumor characteristics for the remaining 91 cases are shown in Table 1, stratified by percentage of monosomy 3. Overall, the mean patient age was 64.7 years (range 20 to 91). Forty-nine patients were male, and 42 patients were female. The mean tumor height was 5.91 mm (range 1.99 to 10.85 mm). The mean number of cells evaluated for FISH analysis was 273 (range 28 to 520). The mean percentage of cells with monosomy 3 was 62.9% (range 4.7% to 100%). Eighteen patients (20%) developed metastatic disease over a mean follow-up period of 28.9 months (range 3 to 76 months).

**Table 1:** Patient and tumor characteristics of 91 cases of uveal melanoma positive for monosomy 3 by FISH from transscleral FNAB.

**Percentage of monosomy 3**	**1%–33%**	**33%–66%**	**66%–100%**	**Overall**
Number of cases	24	15	52	91
Mean age (range)	62.8 (20–91)	64.3 (29–79)	65.7 (39–91)	64.7 (20–91)
Male/Female	15/9	7/8	27/25	49/42
Mean tumor height in mm (range)	5.2 (2.0–10.9)	5.3 (2.3–9.0)	6.4 (2.0–10.8)	5.91 (1.99–10.85)
Number of tumors with ciliary body involvement	5 (20.8%)	3 (20%)	16 (30.8%)	24 (26.4%)
Mean number of cells evaluated (range)	282 (83–520)	265 (48 to 410)	271 (28–483)	273 (28–520)
Number of patients with metastasis	1 (4.2%)	5 (33.3%)	12 (23.1%)	18 (20%)
Mean follow-up in months (range)	29.2 (3–65)	21.3 (5–55)	31.6 (3–76)	28.9 (3–76)
Mean percentage of monosomy 3 (range)	-	-	-	62.9% (4.7%–100%)

**Figure 1:**
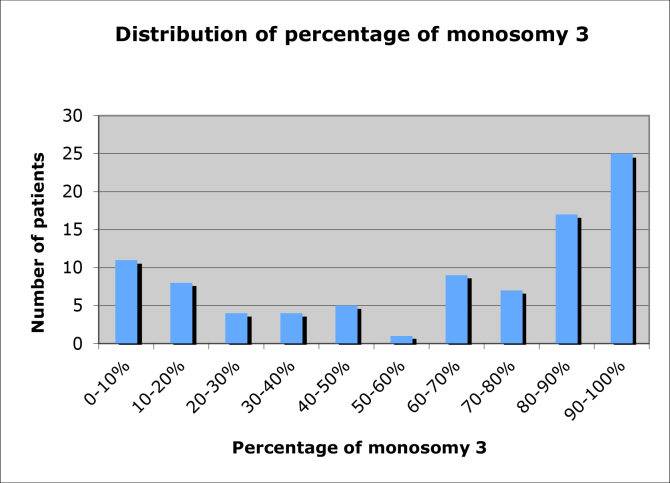
Distribution of percentage of monosomy 3 in 91 patients with uveal melanoma positive for monosomy 3 according to fluorescence in situ hybridization of transscleral fine needle aspiration biopsy specimens.

**Figure 2:**
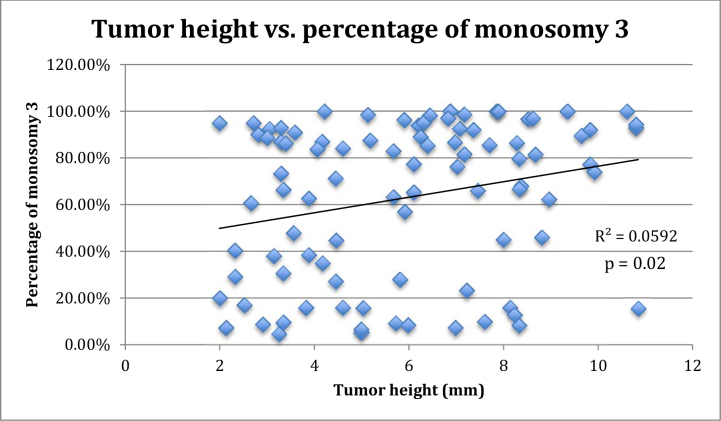
Relationship between tumor height and percentage of monosomy 3 in 91 patients with uveal melanoma positive for monosomy 3 according to fluorescence in situ hybridization of transscleral fine needle aspiration biopsy specimens.

The distribution of the percentage of cells positive for monosomy 3 per patient biopsy sample is shown in Figure 1. The relationship between tumor height and percentage of monosomy 3 per biopsy sample is shown in Figure 2. There was a significant positive correlation between tumor height and percentage of monosomy 3 (p=0.02).

**Figure 3:**
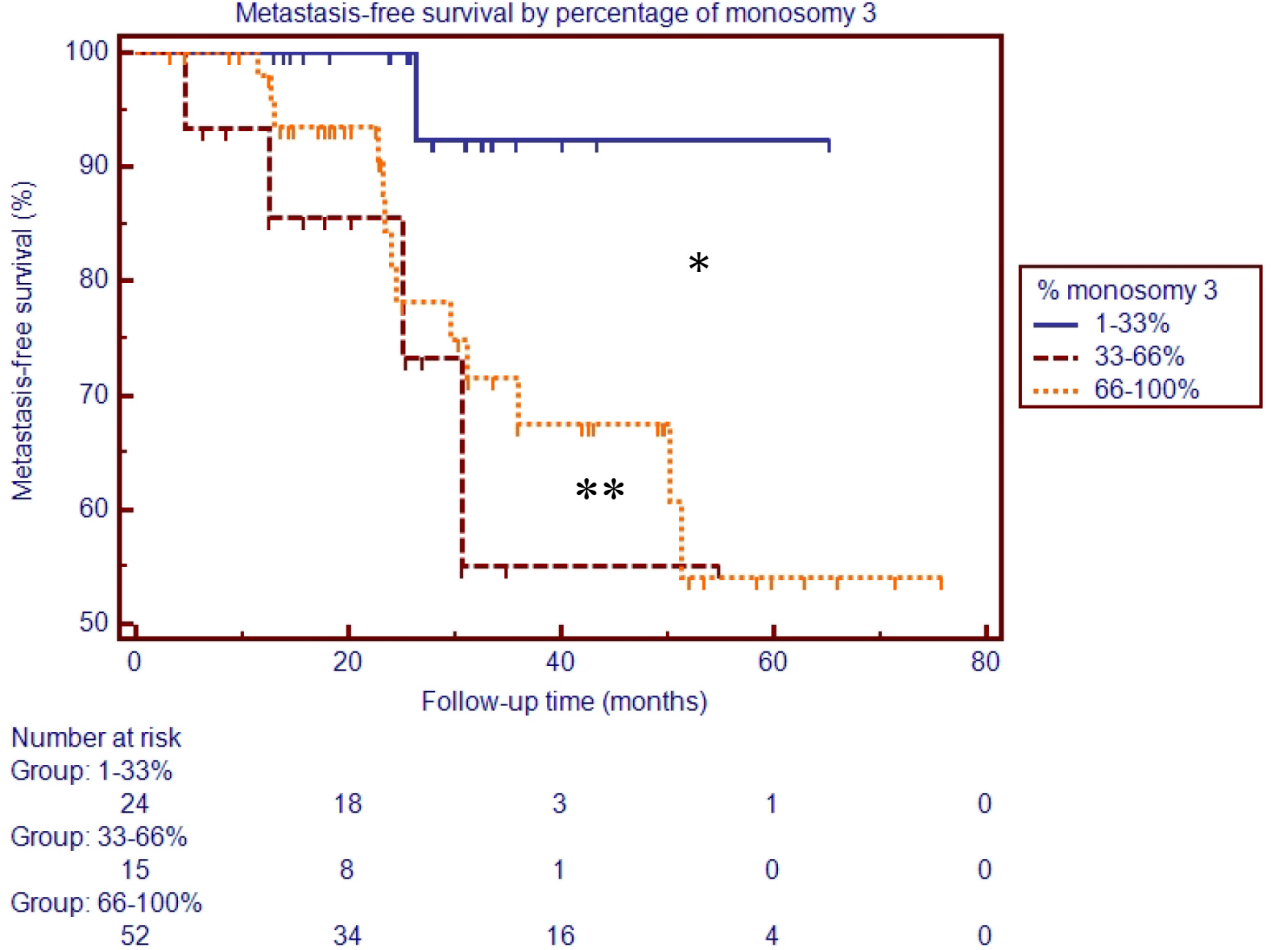
Kaplan–Meier analysis of metastasis-free survival by percentage of monosomy 3.

The Kaplan–Meier survival curve among patients with 1%–33%, 33%–66%, and 66%–100% of monosomy 3 positive cells is shown in Figure 3. There was a significantly lower metastasis-free survival rate in patients who had more than 33% cells positive for monosomy 3 versus patients who had fewer than 33% cells positive for monosomy 3. However, there was no significant difference in the metastasis-free survival rate between patients who had 33%–66% cells positive for monosomy 3 and patients who had 66%–100% cells positive for monosomy 3.

## Discussion

We report on the heterogeneous distribution of monosomy 3 in 91 patients with uveal melanoma who underwent transscleral FNAB during brachytherapy and were positive for monosomy 3 according to FISH. Percentage of monosomy 3 ranged between 4.7% and 100%, with a mean of 62.9%, which is consistent with previous reports of variation in chromosome 3 copy number in uveal melanomas [8-14]. Overall, 18 patients (20%) developed metastatic disease during a mean follow-up interval of 28.9 months. Moreover, we found a significant association between the percentage of monosomy 3 in a biopsy sample and the development of metastatic disease in the patient. The metastasis-free survival rate significantly decreased in patients whose tumors had more than 33% of cells positive for monosomy 3. In addition, there was a significant positive correlation between tumor height and percentage of monosomy 3.

Our study also has important prognostic implications for patients undergoing globe-sparing therapy. The majority of previous reports on the prognostic value of monosomy 3 focused on samples obtained during enucleation [4]. Because tumors that are selected for enucleation are generally larger and more advanced than those treated with brachytherapy, the prognosis for patients with monosomy 3 positive tumors that undergo enucleation may be different from that for patients with monosomy 3 tumors who are candidates for brachytherapy. We report an overall rate of metastasis of 20% over a mean follow-up interval of 28.9 months. This is similar to the metastatic rate reported by Shields et al. [24] in a group of patients with uveal melanomas who underwent FNAB during brachytherapy. The FNAB specimens were analyzed with microsatellite analysis. Among 126 patients with complete monosomy 3, the cumulative probability of metastasis was 8.9% at 24 months and 24.0% at 36 months. The minimum percentage of cells with only one copy of chromosome 3 necessary to categorize a tumor as monosomy 3 positive was not reported.

Our finding that a percentage of monosomy 3 above 33% is associated with significantly decreased metastasis-free survival is similar to the findings of Bronkhorst et al. [8], who performed karyotyping and FISH on cultured cells and FISH on isolated nuclei of enucleated specimens of primary uveal melanoma [8]. The authors found a range of percentages for monosomy 3 in the samples, and evaluated the predictive value of monosomy 3 at various percentages for death by metastatic disease. Monosomy 3 positivity with a cutoff value of 30% was the most robust predictor of death due to metastasis. The authors also found that threshold monosomy 3 percentages of 5% and 50%, in addition to 30%, were associated with significantly increased risk of metastasis-related death.

Our study differs from that of Bronkhorst et al. [8] in that we performed cytogenetic analysis on material obtained with FNAB during brachytherapy, rather than on enucleated specimens. Thus, our finding confirms the prognostic value of monosomy 3 with a cutoff of 30%–33% cells positive for monosomy 3 in FNAB specimens.

Van Den Bosch et al. [16] also evaluated the risk of metastasis-related death according to the percentage of monosomy 3 positive cells in patients who underwent enucleation with FISH analysis of tumor specimens [16]. In that study, risk of death from metastatic disease was analyzed with FISH among patients with tumor specimens harboring 0%–33%, 33%–66%, and 66%–100% cells positive for monosomy 3. There was a statistically significant incrementally worse prognosis in patients with higher percentages of monosomy 3. In our study, we found, similar to Van Den Bosch et al. [16], that patients with 1%–33% cells positive for monosomy 3 had a significantly better metastasis-free survival rate. However, unlike Van Den Bosch et al. [16], we found no difference in prognosis between patients with 33%–66% cells positive for monosomy 3 and 66%–100% cells positive for monosomy 3. Our study results might have been skewed by a single outlier in the 33%–66% cells positive for monosomy 3 group who developed metastasis at 30 months (Figure 3). If our sample size were increased, we might find a statistically significant difference between the 33%–66% and 66%–100% groups, as did Van Den Bosch et al. [16]. Alternatively, the disparity between our results and those of Van Den Bosch et al. [16] may be due to the difference in specimen type (enucleation versus FNAB). Although FISH on FNAB samples has been shown to be an accurate predictor of the presence of monosomy 3 in uveal melanomas [5,6], the percentage of monosomy 3 according to FISH analysis of the FNAB samples may not represent the overall percentage of monosomy 3 cells in the tumor. Because FNAB samples only a portion of a heterogeneous tumor, the material obtained with FNAB is subject to sampling error. In addition, because FISH cannot be used to determine morphology and cell type, stromal cells such as leukocytes and endothelial cells may be included in FISH analysis. Because we lack the cytopathology of FISH samples, we cannot accurately identify the percentage of stromal cells in the FISH analysis.

Several studies have reported that larger tumors are more likely to be positive for monosomy 3 [25,26]. We evaluated the relationship between tumor height and percentage of cells positive for monosomy 3, and we found that larger tumors contained a significantly higher percentage of monosomy 3 cells (Figure 2). We hypothesize that a larger, more advanced tumor might harbor a higher percentage of cells that had the opportunity to lose one copy of chromosome 3 [27,28]. However, smaller tumors may harbor a smaller percentage of monosomy 3 tumor cells due to having been in existence for a shorter period.

We found a bimodal distribution of percentage of monosomy 3 in the samples obtained with transscleral FNAB, with the greatest number of samples containing 80%–100% cells positive for monosomy 3 cells (Figure 1). This differs from the distribution of the percentage of monosomy 3 reported by Bronkhorst et al. [8] in enucleation specimens. The majority of the monosomy 3 cases reported in that study appeared to cluster near 50% [8]. The skewed distribution in our study may be related to sampling error. As discussed previously, the percentage of monosomy 3 in an FNAB sample may not correspond to the percentage of monosomy 3 in the entire tumor. In many cases, the monosomy 3 tumor cells may be physically clustered within a tumor such that when a positive sample is obtained, most of the cells are positive for monosomy 3.

Although monosomy 3 is the strongest cytogenetic predictor of metastatic disease, the mechanism by which loss of chromosome 3 increases risk of metastasis is unclear. The loss of one copy of chromosome 3 is associated with several factors known to portend poor prognosis in uveal melanoma, including ciliary body involvement, extraocular spread, larger basal tumor diameter, epithelioid cells, closed vascular loops, and high mitotic rate [25]. In addition, monosomy 3 is associated with all other cytogenetic alterations in uveal melanomas, particularly abnormalities in chromosomes 1, 6, and 8, and the loss of chromosome 3 is thought to represent the initial cytogenetic aberration necessary for the development of metastatic potential [27-29]. The specific gene or genes on chromosome 3 whose loss leads to the eventual ability to metastasize are only beginning to emerge. Mutations in the BRCA1 associated protein-1 (ubiquitin carboxy-terminal hydrolase) (*BAP1*) gene on chromosome 3p21 may be the reason melanoma cells lose one copy of chromosome 3 [30,31]. Loss of heterozygosity studies have attempted to identify the location of a putative tumor suppressor on chromosome 3, and have identified 3p25 as a region of common allelic loss [32]. Several candidate genes in this region may be important in uveal melanoma metastasis; however, other regions of chromosome 3 also show loss of heterozygosity in other studies [33]. Identifying the specific pathway by which monosomy 3 leads to uveal melanoma metastasis will guide future efforts to develop targeted therapies for this disease.

The strengths of this study include a single center with a standardized CLIA-approved laboratory with cytogeneticists experienced in interpreting and reporting the FISH results of uveal melanoma and an operator experienced in a consistent FNAB technique. Other strengths include excellent patient follow-up and reporting of metastasis. A relative weakness of the study is the relatively short follow-up period for evaluating metastatic outcome. Uveal melanoma metastasis has been reported to occur more than 15 years after diagnosis of the primary tumor [3]. Further follow-up of this cohort is warranted.

In summary, we report the prognostic value, presence, and range of heterogeneity of monosomy 3 in an FNAB sample. Among a group of patients treated with globe-sparing therapy, the 29-month rate of metastatic disease was 20%. Larger tumors were associated with a significantly higher percentage of monosomy 3 in the FNAB sample. Moreover, our results indicate that tumors with more than 33% of cells positive for monosomy 3 in an FNAB specimen have a poor prognosis compared to tumors with lower percentages of monosomy 3. Our findings confirm results from enucleation specimens in FNAB samples and have implications for counseling patients about their disease-related prognosis.

## References

[1] Hu DN, Yu GP, McCormick SA, Schneider S, Finger PT (2005). Population-based incidence of uveal melanoma in various races and ethnic groups.. Am J Ophthalmol.

[2] Diener-West M, Reynolds SM, Agugliaro DJ, Caldwell R, Cumming K, Earle JD, Hawkins BS, Hayman JA, Jaiyesimi I, Jampol LM, Kirkwood JM, Koh WJ, Robertson DM, Shaw JM, Straatsma BR, Thoma J (2005). Development of metastatic disease after enrollment in the COMS trials for treatment of choroidal melanoma: Collaborative Ocular Melanoma Study Group Report No. 26.. Arch Ophthalmol.

[3] Kujala E, Makitie T, Kivela T (2003). Very long-term prognosis of patients with malignant uveal melanoma.. Invest Ophthalmol Vis Sci.

[4] Prescher G, Bornfeld N, Hirche H, Horsthemke B, Jockel KH, Becher R (1996). Prognostic implications of monosomy 3 in uveal melanoma.. Lancet.

[5] Midena E, Bonaldi L, Parrozzani R, Tebaldi E, Boccassini B, Vujosevic S (2006). In vivo detection of monosomy 3 in eyes with medium-sized uveal melanoma using transscleral fine needle aspiration biopsy.. Eur J Ophthalmol.

[6] Naus NC, Verhoeven AC, van Drunen E, Slater R, Mooy CM, Paridaens DA, Luyten GP, de Klein A (2002). Detection of genetic prognostic markers in uveal melanoma biopsies using fluorescence in situ hybridization.. Clin Cancer Res.

[7] McCannel TA, Chang MY, Burgess BL (2012). Multi-Year Follow-up of Fine-Needle Aspiration Biopsy in Choroidal Melanoma.. Ophthalmology.

[8] Bronkhorst IH, Maat W, Jordanova ES, Kroes WG, Schalij-Delfos NE, Luyten GP, Jager MJ (2011). Effect of heterogeneous distribution of monosomy 3 on prognosis in uveal melanoma.. Arch Pathol Lab Med.

[9] Maat W, Jordanova ES, van Zelderen-Bhola SL, Barthen ER, Wessels HW, Schalij-Delfos NE, Jager MJ (2007). The heterogeneous distribution of monosomy 3 in uveal melanomas: implications for prognostication based on fine-needle aspiration biopsies.. Arch Pathol Lab Med.

[10] Meir T, Zeschnigk M, Masshofer L, Pe'er J, Chowers I (2007). The spatial distribution of monosomy 3 and network vasculogenic mimicry patterns in uveal melanoma.. Invest Ophthalmol Vis Sci.

[11] Mensink HW, Vaarwater J, Kilic E, Naus NC, Mooy N, Luyten G, Bruggenwirth HT, Paridaens D, de Klein A (2009). Chromosome 3 intratumor heterogeneity in uveal melanoma.. Invest Ophthalmol Vis Sci.

[12] Sandinha T, Farquharson M, McKay I, Roberts F (2006). Correlation of heterogeneity for chromosome 3 copy number with cell type in choroidal melanoma of mixed-cell type.. Invest Ophthalmol Vis Sci.

[13] Schoenfield L, Pettay J, Tubbs RR, Singh AD (2009). Variation of monosomy 3 status within uveal melanoma.. Arch Pathol Lab Med.

[14] White VA, McNeil BK, Thiberville L, Horsman DE (1996). Acquired homozygosity (isodisomy) of chromosome 3 during clonal evolution of a uveal melanoma: association with morphologic heterogeneity.. Genes Chromosomes Cancer.

[15] Aronow M, Sun Y, Saunthararajah Y, Biscotti C, Tubbs R, Triozzi P, Singh AD (2012). Monosomy 3 by FISH in uveal melanoma: variability in techniques and results.. Surv Ophthalmol.

[16] van den Bosch T, van Beek JG, Vaarwater J, Verdijk RM, Naus NC, Paridaens D, de Klein A, Kilic E (2012). Higher percentage of FISH-determined monosomy 3 and 8q amplification in uveal melanoma cells relate to poor patient prognosis.. Invest Ophthalmol Vis Sci.

[17] Scholes AG, Damato BE, Nunn J, Hiscott P, Grierson I, Field JK (2003). Monosomy 3 in uveal melanoma: correlation with clinical and histologic predictors of survival.. Invest Ophthalmol Vis Sci.

[18] Marathe OS, Wu J, Lee SP, Yu F, Burgess BL, Leu M, Straatsma BR, McCannel TA (2011). Ocular response of choroidal melanoma with monosomy 3 versus disomy 3 after iodine-125 brachytherapy.. Int J Radiat Oncol Biol Phys.

[19] Beran TM, McCannel TA, Stanton AL, Straatsma BR, Burgess BL (2009). Reactions to and desire for prognostic testing in choroidal melanoma patients.. J Genet Couns.

[20] McCannel TA, Burgess BL, Nelson SF, Eskin A, Straatsma BR (2011). Genomic identification of significant targets in ciliochoroidal melanoma.. Invest Ophthalmol Vis Sci.

[21] Young TA, Burgess BL, Rao NP, Glasgow BJ, Straatsma BR (2008). Transscleral fine-needle aspiration biopsy of macular choroidal melanoma.. Am J Ophthalmol.

[22] Young TA, Rao NP, Glasgow BJ, Moral JN, Straatsma BR (2007). Fluorescent in situ hybridization for monosomy 3 via 30-gauge fine-needle aspiration biopsy of choroidal melanoma in vivo.. Ophthalmology.

[23] Chang MY, Kamrava M, Demanes DJ, Leu M, Agazaryan H, Lamb J, Moral JN, Almanzor R, McCannel TA (2012). Intraoperative Ultrasonography-Guided Positioning of Iodine-125 Plaque Brachytherapy in the Treatment of Choroidal Melanoma.. Ophthalmology.

[24] Shields CL, Ganguly A, Bianciotto CG, Turaka K, Tavallali A, Shields JA (2011). Prognosis of uveal melanoma in 500 cases using genetic testing of fine-needle aspiration biopsy specimens.. Ophthalmology.

[25] Damato B, Duke C, Coupland SE, Hiscott P, Smith PA, Campbell I, Douglas A, Howard P (2007). Cytogenetics of uveal melanoma: a 7-year clinical experience.. Ophthalmology.

[26] Shields CL, Ganguly A, Materin MA, Teixeira L, Mashayekhi A, Swanson LA, Marr BP, Shields JA (2007). Chromosome 3 analysis of uveal melanoma using fine-needle aspiration biopsy at the time of plaque radiotherapy in 140 consecutive cases: the Deborah Iverson, MD, Lectureship.. Arch Ophthalmol.

[27] Prescher G, Bornfeld N, Friedrichs W, Seeber S, Becher R (1995). Cytogenetics of twelve cases of uveal melanoma and patterns of nonrandom anomalies and isochromosome formation.. Cancer Genet Cytogenet.

[28] Höglund M, Gisselsson D, Hansen GB, White VA, Sall T, Mitelman F, Horsman D (2004). Dissecting karyotypic patterns in malignant melanomas: temporal clustering of losses and gains in melanoma karyotypic evolution.. Int J Cancer.

[29] Dopierala J, Damato BE, Lake SL, Taktak AF, Coupland SE (2010). Genetic heterogeneity in uveal melanoma assessed by multiplex ligation-dependent probe amplification.. Invest Ophthalmol Vis Sci.

[30] Harbour JW, Onken MD, Roberson ED, Duan S, Cao L, Worley LA, Council ML, Matatall KA, Helms C, Bowcock AM (2010). Frequent mutation of BAP1 in metastasizing uveal melanomas.. Science.

[31] Couturier J, Saule S (2012). Genetic determinants of uveal melanoma.. Dev Ophthalmol.

[32] Parrella P, Fazio VM, Gallo AP, Sidransky D, Merbs SL (2003). Fine mapping of chromosome 3 in uveal melanoma: identification of a minimal region of deletion on chromosomal arm 3p25.1-p25.2.. Cancer Res.

[33] Onken MD, Worley LA, Person E, Char DH, Bowcock AM, Harbour JW (2007). Loss of heterozygosity of chromosome 3 detected with single nucleotide polymorphisms is superior to monosomy 3 for predicting metastasis in uveal melanoma.. Clin Cancer Res.

